# Longitudinal Associations Between Support and Prosocial Behavior Across Adolescence: The Roles of Fathers, Mothers, Siblings, and Friends

**DOI:** 10.1007/s10964-023-01885-5

**Published:** 2024-01-20

**Authors:** Marije van Meegen, Jolien Van der Graaff, Gustavo Carlo, Wim Meeus, Susan Branje

**Affiliations:** 1 Department of Youth and Family, Utrecht University, Utrecht, The Netherlands; 2 Department of Education, University of California, Irvine, CA USA

**Keywords:** Prosocial behavior, Adolescence, Family members, Friends, Support, Socialization

## Abstract

Family members and friends can play an important role in adolescents’ prosocial behavior. To better understand the relation between support and prosocial behavior in adolescence, it’s important to conduct longitudinal studies that distinguish between within-dyad variance and between-dyad variance. The current study investigated longitudinal associations between adolescents’ prosocial behavior, autonomy support, and emotional support from family and friends across adolescence. Across six annual years, 497 Dutch adolescents (284 boys; mean age T1 = 13.03 years, SD_age_ = 0.46), fathers, mothers, siblings, and friends reported on their prosocial behavior. Adolescents also reported on perceived autonomy and emotional support. Between-dyads almost all associations of support and prosocial behavior of family members and friends with adolescents’ prosocial behavior were significant, with higher levels of adolescents’ prosocial behavior being associated with higher levels of prosocial behavior and support from fathers, mothers and friends. Within-dyads, several concurrent associations were significant, but within-dyads links between prosocial behavior and autonomy support are particularly driven by adolescent-mother or adolescent-sibling effects. This study highlights processes that occurred either at the between-dyad level or at the within-dyad level, but that varied per relationship type and that adolescents are the main catalysts in within-dyads changes in prosocial behavior and support. Preregistration: This study was preregistered on 20 January 2020 at https://osf.io/vxkm3/?view_only=dca87fd1585c444ba5cd5a00c22280ae.

## Introduction

Adolescence is an important period for developing prosocial behaviors or voluntary behaviors to benefit others (Van der Graaff et al., 2018). Prosocial behaviors play an important positive role in adolescents’ interpersonal relationships (Carlo, 2014). Close relationships have the most pronounced influence on adolescents’ social development, and close relationships with parents, siblings and friends have been found to contribute to adolescents’ prosocial behavior (Eisenberg et al., 2015). Parents, siblings and friends can promote adolescents’ prosocial behavior in two ways: by modeling prosocial behavior and by providing a warm and supportive relational context (Eisenberg et al., 2015). Social learning theory posits that adolescents learn prosocial behaviors by observing and mimicking others’ behaviors, particularly in close relationships (Hoffman, 2008). In addition, high-quality and supportive relationships with family members and friends may increase the internalization of prosocial standards and behaviors (Hastings et al., 2007). When adolescents have secure bonds with family members and friends and feel supported and accepted by others, they have the resources to attend to others’ needs and display prosocial behaviors (Shaver et al., 2019). Conversely, engaging in prosocial behavior might also facilitate high-quality relationships (e.g., Padilla-Walker et al., 2015a). The main aim of this study was to understand the longitudinal associations of autonomy support, emotional support, and prosocial behavior of fathers, mothers, siblings, and friends to adolescents’ prosocial behavior. We examined these processes both at the between-dyad level and at the within-dyad level.

### The Role of Prosocial Behavior From Family and Friends in Adolescents’ Prosocial Behavior

From a social learning perspective, it is assumed that when relational partners model or talk about prosocial behaviors, adolescents are more likely to display these behaviors (Hoffman, 2008). Relationships with parents, siblings, and friends are important to consider in this regard. Parents are important role models for their children. They are considered as the primary socializers of children’s prosocial behavior, as they lay the foundations for their psychosocial functioning within the norms and standards of society (Hastings et al., 2007). In general, relationships with siblings and friends are to a much larger extent based on reciprocity and differ from relationships with parents (Branje et al., 2002). Therefore, these relationships offer a unique context to model and observe each other’s prosocial behaviors. Sibling relationships are involuntary and can be high in support and conflict but they generally last no matter what. In addition, adolescents in general spend more time with their siblings than with their parents (Updegraff et al., 2011), which also underlines the strong impact they can have on each other’s prosocial behaviors (McHale et al., 2012). Sibling relationships thus might serve as context to try out different behaviors without risking ending the relationship (Buist et al., 2013). In contrast, friendships are voluntary relationships and can dissolve at any time (Furman & Rose, 2015), therefore adolescents are more motivated to show prosocial behavior in high-quality friendships to maintain these relationships. Thus, interactions with both siblings and friends are considered important training grounds for prosocial behaviors.

Previous research indeed showed some support for positive associations between prosocial behavior from parents, siblings and friends with adolescents’ prosocial behavior (Carlo, 2014; Eisenberg & Valiente, 2002). A cross-sectional study showed that when parents discuss and engage adolescents in prosocial activities, their adolescent children exhibit more prosocial behaviors (Carlo et al., 2007). Relatedly, parents who adhered to higher levels of implicit rules (unwritten rules and norms within the family such as “make decisions together as a family,” p. 72, Crane et al., 2020) on prosociality in family processes had adolescents who showed more prosocial communication with both parents (Crane et al., 2020). Regarding siblings, there is empirical evidence for positive associations between siblings’ prosocial behavior during childhood. A short-term longitudinal study with 4 and 9 year old children showed positive within-time associations and positive cross-lagged effects between siblings’ prosocial behavior (Pike & Oliver, 2017). Results of this study thus suggest that there are positive associations between siblings’ prosocial behavior in childhood. As for friends, longitudinal studies that were conducted in classrooms showed that adolescents with peers who engage in prosocial behavior are more likely to show prosocial behavior themselves (e.g., Laninga-Wijnen et al., 2020).

In sum, the empirical evidence for modeling effects is growing and typically shows positive associations between prosocial behavior from parents, siblings, and friends with adolescents’ or children’s prosocial behavior. However, most studies employ a short-term longitudinal design and focus on one specific socialization agent. Furthermore, most research is conducted in childhood, instead of investigating modeling effects from family members and friends across adolescence. Also, as it is assumed that processes that foster prosocial behaviors occur within a person or within dyads, it is crucial to investigate these processes while separating within-dyad effects from between-dyad effects.

### The Role of Support in Adolescents’ Prosocial Behavior

Attachment theorist suggests associations between relationship quality and children’s prosocial behaviors (Wong et al., 2021). When adolescents have a sense of secure bonds with family members and friends and feel supported and accepted by them (Allen, 2008; Pascuzzo et al., 2013), they have the resources to attend to others’ needs and display prosocial behaviors (Shaver et al., 2019). Further, according to Self Determination Theory scholars, social behaviors are best internalized when the basic needs for autonomy and relatedness are met in close relationships (Deci & Ryan, 2000). These needs can be met through autonomy support and emotional support. Autonomy support is reflected by tolerating, accepting and respecting adolescents’ opinions, feelings and ideas (Shulman et al., 1997), and emotional support is characterized by showing warmth, acceptance, and empathy (Miklikowska et al., 2011). In contexts where adolescents feel supported and respected, this might promote positive psychological functioning, such as prosocial behaviors (Shaver et al., 2019). Moreover, high levels of consistent social support offer the opportunity to gain strong moral and social bond wherein adolescents show prosocial behaviors (Colvin et al., 2002).

The evidence concerning the links between parental autonomy and adolescents’ prosocial behavior mostly stems from cross-sectional studies, and only to a limited extent from longitudinal studies. Autonomy support and emotional support are central constructs that characterize parenting, which is in line with Baumrind’s theory (1991) who identifies the parenting dimensions of demandingness and responsiveness. Authoritative parents are both responsive and demanding, which means that they are warm and responsive to adolescents’ needs, offer clear rules, and expectations while encouraging autonomy. A two-year longitudinal study showed bidirectional relations between prosocial behavior and authoritative parenting during adolescence, with the most consistent evidence for the role of adolescents’ behavior on subsequent parenting (Padilla-Walker et al., 2012). This may indicate that parents, in general, are more supportive and autonomy granting toward adolescents who show prosocial behavior. Relatedly, a meta-analysis showed that parental autonomy support is one of the key parenting behaviors that is positively related to adolescents’ prosocial behaviors with stronger effect sizes for cross-sectional studies than for longitudinal studies (Wong et al., 2021). Furthermore, two cross-sectional studies showed that parental autonomy support was positively associated with adolescents’ prosocial behavior (Lan et al., 2019; Wong et al., 2022). As cross-sectional studies cannot address the direction of effects, the current study investigates these links longitudinally across adolescence to disentangle whether parental autonomy support predicts adolescents’ prosocial behavior or vice versa.

Regarding emotional support, previous research revealed positive concurrent associations of emotional support from parents with adolescents’ prosocial behavior (e.g., Laible, 2007). Longitudinal findings, however, are mixed. One study, in adolescents aged 10–14 years, showed that maternal connectedness (i.e., emotional support) positively predicted adolescents’ prosocial behavior, while paternal connectedness does not predict adolescents’ prosocial behavior one year later (Padilla-Walker & Christensen, 2011). One study found for adolescents between 9 to 14 years old that maternal warmth was positively related to adolescents’ prosocial behavior one year later across three successive waves (Carlo et al., 2010). In addition, in adolescents with a mean age of 12 years both maternal warmth and paternal warmth were related to adolescents’ prosocial behavior one year later (Padilla-Walker et al., 2016). However, another longitudinal study showed that a combined score of paternal and maternal warmth was not predictive of adolescents’ prosocial behavior between age 13 and 18 years (Lee et al., 2017). The discrepancies in findings of previous longitudinal studies might be because these studies examined different periods of adolescence and did not all investigate emotional support from mothers and fathers separately. Therefore, the current study examined the roles of mothers’ and fathers’ emotional support in adolescents’ prosocial behavior systematically across adolescence.

Regarding the different roles of mothers and fathers in the socialization of prosocial behavior, previous research in childhood revealed that both parents contributed to prosocial development, but longitudinal associations of parental support to children’s prosocial behavior were stronger for mothers than for fathers (Hastings et al., 2015). This might be due to mothers being more involved in childrearing activities than fathers and therefore having more opportunities to promote their children’s prosocial behavior (Hastings et al., 2015). However, Self Determination Theory posits that parental influence is equal across parents, independent of parent or child gender (Deci & Ryan, 2000), and in line with this a meta-analysis, mainly based on cross-sectional studies, found that associations of mothers’ and fathers’ parenting with adolescents’ prosocial behavior were as strong (Wong et al., 2021). Moreover, the potentially different roles of fathers and mothers in adolescents’ prosocial behavior have not often been studied longitudinally, as most research used combined scores of support from both parents or only included scores from mothers. Therefore, in the current study, we explored the unique contributions of support from both mothers and fathers to adolescents’ prosocial behavior.

Evidence on the role of support from siblings and friends in adolescents’ prosocial behavior is also scarce and inconsistent. The role of autonomy support from siblings in adolescents’ prosocial behavior has not been studied. Regarding autonomy support from friends, it was found that autonomy support from classmates was positively related to adolescents’ prosocial behavior (Ma et al., 2022). Moreover, longitudinal research revealed that higher levels of control in friendships were associated with lower adolescents’ prosocial behavior (e.g., Padilla-Walker et al., 2015b). For emotional support, affection from siblings was positively associated with adolescents’ prosocial behavior and positively predicted later adolescent prosocial behavior (Harper et al., 2016). Results for emotional support in friendships are inconclusive; one study showed that affective bonds with friends were not significantly related to adolescents’ prosocial behavior (Carlo et al., 2012), another study showed that strong affective bonds between friends were longitudinally associated with higher levels of adolescents’ prosocial behaviors, but only indirectly via adolescents’ sympathy (e.g., Padilla-Walker et al., 2015b). Thus, evidence for positive associations of prosocial behavior with autonomy support from siblings and friends is scarce. Moreover, emotional support from siblings appeared to be positively related to prosocial behavior (Harper et al., 2016), but for friends, these associations are inconsistent.

### The Role of Adolescents’ Prosocial Behavior in Interpersonal Relationships

Different theoretical models stress that the relationships between individuals are transactional and reciprocal (e.g., Bell, 1977). Comparable mechanisms might operate in prosocial behavior. Research has for example shown that parents and children reinforce each other’s negative behavior, setting up a cycle of coercion where children elicit particular types of responses from their parents and where parents’ behavior induces children to behave in particular ways (Patterson, 2002). Similarly, when adolescents show prosocial behavior, parents, siblings, and friends may positively reinforce this by providing autonomy support, emotional support and prosocial behavior. This may set up a cycle of prosocial behavior; adolescents elicit positive responses from parents, siblings and friends and this reinforces adolescents’ prosocial behavior (Dishion et al., 1992). This is particularly evident for siblings (e.g., Defoe et al., 2013) and friends (e.g., Piehler & Dishion, 2007), as adolescents have similar relational status roles in these relationships, but adolescents can also affect their parents’ behavior (e.g., Bell, 1977). Hence, increases in adolescents’ prosocial behavior may also facilitate prosocial behaviors from parents, siblings and friends. Similarly, adolescents’ prosocial behavior is also expected to be predictive of autonomy support and emotional support provided by parents, siblings, and friends. Family and friends of adolescents who display increases in prosocial behaviors may respond to these actions by being more autonomy and emotionally supportive. It is likely easier to be supportive when adolescents often engage in positive and responsible behaviors.

Empirical researchers have, indeed, found that adolescents who reported more prosocial behaviors subsequently perceived higher levels of maternal emotional support (Carlo et al., 2010) and parental acceptance (Bornstein et al., 2018) than adolescents who reported less prosocial behavior. Likewise, higher levels of adolescents’ prosocial behavior towards friends predicted an increase in friendship quality (Meuwese et al., 2017). Moreover, prosocial behaviors toward friends positively predicted how connected adolescents felt with their friends (Padilla-Walker et al., 2015a). Although this research suggests that engaging in prosocial behavior may facilitate supportive relationships, the results were from studies that did not separate between-dyad level associations from within-dyad level associations. By examining these processes both at the between-dyad level and at the within-dyad level, we not only addressed if adolescents whose relational partners reported more autonomy and emotional support also reported more prosocial behavior, but also whether fluctuations in autonomy and emotional support within the dyads predicted fluctuations in adolescents’ prosocial behavior.

### Gender Differences

Gender intensification theory posits that there is increased socialization pressure to conform to gender roles during adolescence (Hill & Lynch, 1983). Girls are sensitive to supportive behaviors from others and are expected to show more nurturing and caring behaviors (Hastings et al., 2007). Boys are more independent and adhere to more masculine types of prosocial behavior (Xiao et al., 2019). Therefore, girls might be more sensitive to prosocial behaviors and support from others and therefore the associations between support and prosocial behavior might be stronger for girls than for boys in adolescence.

Gender differences in mean levels of prosocial behavior, with girls scoring higher than boys, are well-established in the literature (e.g., Van der Graaff et al., 2018). In addition, consistent with gender intensification theory, these gender differences in prosocial behaviors are stronger in adolescence than in childhood (Eisenberg & Fabes, 1998). However, there is less evidence for gender differences in the links of parents’, siblings’ and friends’ prosocial behavior or support to adolescents’ prosocial behavior. Meta-analyses found no gender differences in the links between parenting (i.e., autonomy support and emotional support) and prosocial behaviors (Wong et al., 2021) and between relationship quality with family and peers and empathy (which is related to prosocial behavior) (Boele et al., 2019).

## Current Study

Previous research revealed inconsistent evidence for the longitudinal associations between prosocial behavior, autonomy support, emotional support from family and friends and adolescents’ prosocial behavior within dyads. The current study examined within-dyad associations and lagged effects of adolescents’ prosocial behavior with prosocial behavior, autonomy support, and emotional support of mothers, fathers, siblings, and friends using six waves of longitudinal data spanning a period of five years.

The first hypothesis was that adolescents’ prosocial behavior, perceived autonomy support (Hypothesis 1a), perceived emotional support (Hypothesis 1b) and prosocial behavior (Hypothesis 1c) from fathers, mothers, siblings and friends were positively associated at the between-dyad level. The second hypothesis was that adolescents’ prosocial behavior and perceived autonomy support (Hypothesis 2a), perceived emotional support (Hypothesis 2b), and prosocial behavior (Hypothesis 2b), from fathers, mothers, siblings and friends positively associated at the within-dyad level. The third hypothesis was that changes in adolescents’ prosocial behavior predicted changes in prosocial behavior (Hypothesis 3a), perceived autonomy support (Hypothesis 3b) and emotional support (Hypothesis 3c) from family and friends one year later at the within-dyad level. The fourth hypothesis was that changes in prosocial behavior (Hypothesis 4a), perceived autonomy support (Hypothesis 4b), and perceived emotional support (Hypothesis 4c) from family and friends predicted changes in adolescents’ prosocial behavior one year later at the within-dyad level. No firm hypotheses were formulated for adolescent gender differences in the associations between adolescents’ prosocial behavior and prosocial behavior and autonomy support and emotional support from parents, siblings and friends. The fifth hypothesis was that support from both fathers and mothers would positively predict adolescents’ prosocial behavior, but that effects would be stronger for mothers-adolescent dyads than for father-adolescent dyads.

## Methods

### Participants and Procedure

The current study used six annual waves of questionnaire data from the ongoing longitudinal Research on Adolescent Development and Relationships - Young project (RADAR-Y). The dataset can be found on Data Archiving and Networked Services (DANS) (link: 10.17026/dans-zrb-v5wp). The study followed 497 Dutch adolescents (284 boys, *M*_age_ = 13.03, SD_age_ = 0.46), their fathers (*n* = 446, *M*_age_ = 46.74, SD_age_ = 5.10), their mothers (*n* = 495, *M*_age_ = 44.40, SD_age_ = 4.45), their siblings (*n* = 417 45.7% girls, *M*_age_ = 14.75, SD_age_ = 3.11), and their friends (*n* = 449, 39.4% girls, *M*_age_ = 13.18, SD_age_ = 0.80). Based on parental job earnings (T1), the majority of these youngsters came from medium or high (87.70%) social-economic status. Most participants lived with both their parents (84.7%). The rest of the participants lived in other family compositions, such as with their biological parent and their stepparent (5.3%). Of the participants, 94.8% identified themselves as native Dutch and 5.2 % as an ethnic minority.

Adolescents came from four large cities in the center and western parts of the Netherlands. Before the start of the study, families received written information about the study. In each wave, adolescents were asked to nominate their friends and provide their friends’ contact information, and these friends were invited to participate in the study. The RADAR project set out to involve 500 families, each including an adolescent, both parents, one sibling between the ages of 10–20 years, and a friend of the adolescent. Only one sibling could take part, and the adolescents were asked to nominate a friend for the study. Out of all the pairs of siblings, 26.6% were younger and 60.4% were older than the target adolescent. Around 13.1% of the siblings’ scores were missing, regarding friendships, over the course of six waves, between 61.6 and 69.8% of the adolescents nominated the same friend for two successive waves, indicating that their friendships were fairly stable, and 34.9% of the adolescents brought the same best friend to all five waves.

All participants gave written informed consent before the first home visit. Trained research assistants visited participants at 1-year intervals. During these home visits, questionnaires were filled out by family members and friends about their relationships and psychosocial functioning. Participants received 20 euros each for each time data was collected. The Medical Ethical Committee of Utrecht University Medical Centre has approved the RADAR study. Attrition was low: from the sample of the first wave, 85.7% of adolescents, 75.5% of fathers, 84.5% of mothers, 72.4% of siblings, and 74.4% of friends still participated in the last wave.

Attrition analyses revealed that the group of adolescents that dropped out showed fewer prosocial behaviors compared to adolescents that still participated in the study, *t*(82.47) = −2.06*, p* *=* 0.042. They perceived less autonomy support from their mothers, *t*(491) = −2.02*, p* *=* 0.044, and their siblings, *t*(483) = −2.22*, p* *=* 0.027, less emotional support from their fathers, *t*(463) = −3.12*, p* *=* 0.002, and their siblings, *t*(434) = −2.04*, p* *=* 0.042. There were no significant differences in the number of boys or girls between dropouts and stayers *χ*^2^(1) = 0.79, *p* = 0.375. Adolescents that stayed in the study came more often from medium and high SES, *χ*^2^(1) = 11.76, *p* = 0.001, and were older, *t*(84.23) = 2.01*, p* *=* 0.048 compared to the group that dropped out. To assess the missingness in the sample, we conducted Little’s missing completely at random (MCAR) tests. Little’s MCAR tests were nonsignificant and showed that the pattern of missing was at random, with a normed chi-square (*χ*^2^/*df*) of 1.15. To account for the missing data, we used Full Information Maximum Likelihood, with Robust standard errors.

### Measures

#### Prosocial behavior

Adolescents, parents, siblings, and friends reported their own prosocial behavior with the 11-item subscale “prosocial behavior” from the Dutch version of the Self-report of Aggression and Social Behavior Measure (Morales & Crick 1998; Linder et al. 2002). Items were rated on a 7-point Likert scale ranging from 1 (not at all true) to 7 (very true). A sample item is: “I am willing to help others.” For each wave the items were averaged to compute separate mean composite scores for fathers, mothers, siblings, friends and adolescents. Cronbach’s alpha reliabilities ranged from *α* = 0.90 to *α* = 0.93 for adolescents’ reports, for maternal reports from *α* = 0.82 to *α* = 0.87, for paternal reports from *α* = 0.80 to *α* = 0.91, for sibling reports from *α* = 0.88 to *α* = 0.92, and for friend reports from *α* = 0.88 to *α* = 0.92.

#### Perceived autonomy support

Adolescents reported perceived autonomy support from mothers, fathers, siblings, and friends, for each relationship separately. This scale measured to what degree adolescent felt that fathers, mothers, siblings and friends respected their wishes, opinions and needs. The 7-item subscale “balanced relatedness” of the Dutch version of Adolescent Intimacy Revisited (Shulman et al., 1997). For each wave the items were averaged to compute separate mean composite scores for fathers, mothers, siblings, and friends. The items were rated on a 4-point scale ranging from 1(strongly disagree) to 4 (strongly agree). An example item is: “My father/mother/sibling/friend respects my ideas. Construct validity was supported by several studies that used the balanced relatedness scale as an indicator of autonomy support and examined associations with adolescents’ problem behaviors (e.g., Van der Giessen et al., 2014). The results of those studies indicated that higher scores of autonomy support were related to higher scores on sense of family belonging (Rejaän et al., 2022), and adolescents’ educational identity (van Doeselaar et al., 2016). Moreover, lower scores on autonomy support were related to more internalizing and externalizing problems (Vrolijk et al., 2020) and depressive symptoms (van der Giessen et al., 2014). Cronbach’s alphas reliabilities of the autonomy support scale ranged across six waves for reports about mothers from *α* = 0.85 to *α* = 0.89, about fathers from *α* = 0.78 to *α* = 0.85, about siblings from *α* = 0.83 to *α* = 0.89, and about friends from *α* = 0.87 to *α* = 0.92.

#### Perceived emotional support

Adolescents reported on perceived emotional support from fathers, mothers, siblings, and friends for each relationship separately. The 8-item subscale “support” from the Dutch version of the Network of Relationships Inventory (Furman & Buhrmester, 1985) was used. The items were rated on a 5-point scale ranging from 1 (little or no) to 7 (more is impossible). The questionnaire tapped into emotional support measures, for example, “How much does your father/mother/sibling/friend care about you?” For each wave the items were averaged to compute separate mean composite scores for fathers, mothers, siblings, and friends. The psychometric properties of this instrument have been shown to be good (Furman & Buhrmester, 2009). In this study, Cronbach’s alphas reliabilities of the support scale ranged across six waves for reports about mothers from *α* = 0.78 to *α* = 0.85, about fathers from *α* = 0.81 to *α* = 0.88, about siblings from *α* = 0.84 to *α* = 0.86, and about friends from *α* = 0.86 to *α* = 0.89.

### Statistical Analyses

**Fig. 1:**
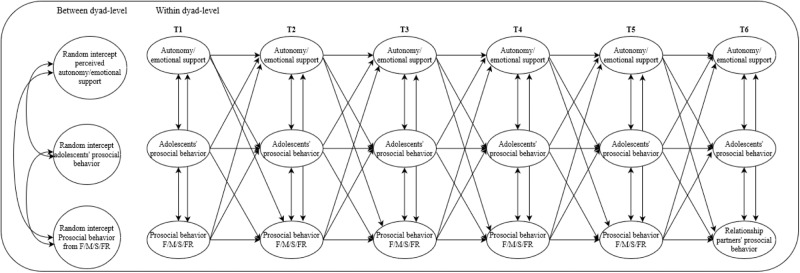
Conceptual model for the links between support and prosocial behavior from fathers, mothers, siblings and friends and adolescents’ prosocial behavior. The figure presents the conceptual model in general. This model was conducted separately for autonomy support, for emotional support, and for fathers, mothers, siblings, friends

The analytic plan was preregistered in the Open Science Framework on 2020-01-20; see anonymized link: https://osf.io/vxkm3/?view_only=dca87fd1585c444ba5cd5a00c22280ae. Random intercept cross-lagged panel models (RICLPM; Hamaker et al., 2015) were used to analyze the longitudinal bidirectional relations of prosocial behavior, autonomy support, and emotional support from fathers, mothers, siblings and friends with adolescents’ prosocial behavior (See Fig. 1). This resulted in separate models (8 final models) for each relationship type and for emotional support and autonomy support to prevent multicollinearity.

Each model consisted of six repeated measures (one for each assessment wave) of adolescents’ prosocial behavior, perceived autonomy support or perceived emotional support, and prosocial behavior from fathers, mothers, siblings and friends. These scores were treated as manifest variables, because adding additional latent scores would increase model complexity. In each model, three latent variables were added, which reflected the three random intercepts of adolescents’ prosocial behavior, perceived autonomy or emotional support, and prosocial behavior from fathers, mothers, siblings and friends. These random intercepts contain all the between-person variance and reflect stable differences between persons. Between-level associations reflect average differences between dyads, and thus show whether average differences in prosocial behavior and support from parents, siblings and friends are associated to differences in adolescents’ prosocial behavior. For each of the six observed repeated measures per construct, six latent variables were added to capture the deviation from the person’s mean. The variance of the observed scores was constrained to zero to ensure that the variance was reflected by either the between- or within-level. Moreover, all within-time correlations between all constructs, stability paths for all constructs, and all possible cross-lagged effects were added. Within-dyad associations between prosocial behavior and support from fathers, mothers, siblings, and friends at the same time reflect correlated change and indicate whether higher or lower levels of prosocial behavior or support are associated with higher or lower levels of adolescents’ prosocial behavior at the same time. Within-dyad lagged effects indicate whether prosocial behavior or support from fathers, mothers, siblings or friends predicts changes in adolescents’ prosocial behavior later in time. Thus, the models yielded between-person correlations, within-time correlations, and within-dyad lagged effects.

In the baseline model, all paths were estimated freely. In subsequent models, paths were constrained across time using a stepwise procedure by first, constraining within-person stabilities and second, by constraining the cross-lagged effects. After each step, the model fit was compared to the fit of the unconstrained baseline model. To assess the overall model fit, we used Root Mean-Square Error of Approximation (RSMEA; ≤0.08), Comparative Fit Index (CFI; ≥0.90), and Tucker-Lewis Index (TLI ≥ 0.90) (Pituch & Stevens, 2016). Model comparisons were evaluated with Satorra and Bentler’s scaled chi-square difference tests (S-B*χ*^2^; Muthen & Muthen, 1998–2017). Based on the model fit indices, we selected the most parsimonious model as the final model.

Moreover, multigroup analyses were conducted to explore whether adolescents’ gender moderated the links between adolescents’ prosocial behavior and perceived support and prosocial behavior from fathers, mothers, siblings and friends. This was done by testing in a stepwise procedure whether model fit improved when parameters were allowed to vary between boys and girls. First, within-time correlations between perceived autonomy support or perceived emotional support and adolescents’ prosocial behavior and within-time correlations between mothers/fathers/siblings/friends’ prosocial behavior and adolescents’ prosocial behavior were released. Second, the cross-lagged paths between autonomy/emotional support, mothers/fathers/siblings/friends’ prosocial behavior, and adolescents’ prosocial behavior were released. Third, between-dyad correlations between perceived autonomy or emotional support, mothers/fathers/siblings/friends’ prosocial behavior, and adolescents’ prosocial behavior were released. Each step was tested against the multigroup baseline model, in which all parameters were constrained to be equal for boys and girls. All data manipulations and measures used during analyses were reported in the current study.

## Results

The final models had an acceptable fit, with indices for RMSEA ranging between 0.01 and 0.06, for CFI between 0.89 and 0.99, and TLI between 0.89 and 0.99 (Table 1). Stepwise constraining the stability paths indicated that constraining the stability paths of adolescents’ prosocial behavior in all different final models (ranges Δ*χ*^2^_SB_(4) = 11.89, *p* = 0.018; Δ*χ*^2^_SB_(4) = 15.89, *p* = 0.003), siblings prosocial behavior (Δ*χ*^2^_SB_(4) = 23.37, *p* = 0.001), fathers’ emotional support (Δ*χ*^2^_SB_(4) = 13.84, *p* = 0.009), mothers’ emotional support (Δ*χ*^2^_SB_ (4) = 11.70, *p* = 0.012), and friends’ autonomy support (Δ*χ*^2^_SB_ (4) = 11.91, *p* = 0.018) worsened model fit (Table 2). Therefore, those paths were not constrained. Stepwise constraining the cross-lagged paths indicated that constraining the paths from mothers’ autonomy support to mothers’ prosocial behavior (Δ*χ*^2^_SB_ (4) = 29.72, *p* = 0.046; Table S1), and the within-time association of siblings’ prosocial behavior with siblings’ emotional support (Δ*χ*^2^_SB_ (4) = 22.70, *p* = 0.001) worsened model fit; thus, those paths were not constrained. All other within-time associations, stability paths and cross-lagged paths could be constrained to be time-invariant.

**Table 1:** Overview model fit indices for all RI-CLPMs

	*χ*^2^ (df)	RMSEA	CFI	TLI
Father–adolescent autonomy support	177.973 (df:128)	0.03	0.97	0.97
Father–adolescent emotional support	176.696 (df:124)	0.03	0.98	0.97
Father–adolescent autonomy support: Multigroup^a^	351.775 (df:279)	0.03	0.97	0.96
Father–adolescent emotional support: Multigroup^a^	338.528 (df:276)	0.03	0.97	0.97
Mother–adolescent autonomy support^a^	192.368 (df:124)	0.03	0.96	0.95
Mother–adolescent emotional support^a^	209.211 (df:124)	0.04	0.96	0.95
Mother–adolescent autonomy support: Multigroup	372.928 (df:279)	0.04	0.95	0.94
Mother–adolescent emotional support: Multigroup	405.164 (df:277)	0.04	0.94	0.94
Sibling–adolescent autonomy support^a^	257.280 (df:88)	0.05	0.91	0.89
Sibling–adolescent emotional support	275.297 (df:120)	0.05	0.93	0.91
Sibling–adolescent autonomy support: Multigroup	455.455 (df:277)	0.05	0.89	0.89
Sibling–adolescent emotional support: Multigroup^a^	486.54 (df:277)	0.06	0.91	0.90
Friend–adolescent autonomy support^a^	128.946 (df:124)	0.01	0.99	0.99
Friend–adolescent emotional support^a^	153.130 (df:128)	0.02	0.98	0.98
Friend–adolescent autonomy support: Multigroup	322.264 (df:278)	0.03	0.92	0.91
Friend–adolescent emotional support: Multigroup	342.565 (df:278)	0.03	0.94	0.94

*χ*^*2*^ chi-square, *df* degrees of freedom, *RMSEA* root-mean-square error of approximation, *CFI* comparative fit index, *TLI* Tucker-Lewis index

^a^Indicate the models that best fitted the data

**Table 2:** Stability paths for prosocial behavior from fathers, mothers, siblings and friends, adolescents’ prosocial behavior, autonomy support and emotional support

Model	Father model			Mother model			Sibling model			Friend model		
	*B* (SE)	*β* (SE)	*p*	*B* (SE)	*β* (SE)	*p*	*B* (SE)	*β* (SE)	*p*	*B* (SE)	*β* (SE)	*p*
Stability paths T1–T6												
Prosocial behavior F/M/S/FR → Prosocial behavior F/M/S/FR	−0.01 (0.05)^a^	−0.01 (0.05)	0.906	0.12 (0.05)^a^	0.10 (0.05)	**0.044**	0.20 (0.06)/ 0.08 (0.06)/ −0.47 (0.10)/−0.35 (0.21)/0.13 (0.21)	0.24 (0.07)/0.11(0.09)/−0.43 (0.12)/−0.54 (0.21)/0.07 (0.11)	**0.001/**0.219/**0.000/0.010**/0.539	0.11 (0.03)^a^	0.11 (0.03)	**0.001**
Prosocial behavior A → Prosocial behavior A	0.22 (08)/0.07 (0.05)/0.19 (0.07)/0.05 (0.07)/0.35 (0.12)	0.21 (0.08)/ 0.08 (0.05)/ 0.19 (0.06)/ 06 (0.08)/ 0.30 (0.11)	**0.006**/ 0.151/ **0.003**/ 0.489/ **0.006**	0.22 (0.08)/ 0.06 (0.05)/ 0.19 (0.07)/ 0.06 (0.06)/ 0.35 (0.12)	0.21 (0.08)/0.07 (0.05)/0.19 (0.06)/0.07 (0.07)/0.31 (0.11)	**0.007/**0.183/**0.00**/0.346/**0.006**	0.22 (0.08)/0.07 (0.05)/0.17 (0.07)/0.05 (0.07)/0.35 (0.12)	0.21 (0.08)/0.08 (0.06)/0.17 (0.06)/0.06 (0.07)/0.31 (0.11)	**0.008/**0.147/**0.007**/0.439/**0.006**	0.21 (0.08)/0.05 (0.05)/0.17 (0.06)/0.07 (0.06)/0.36 (0.12)	0.21 (0.08)/0.06 (0.05)/0.17 (0.06)/0.08 (0.07)/0.31 (0.11)	**0.009**/0.300/**0.004**/0.280/**0.005**
Autonomy support → Autonomy support	0.28 (0.06)	0.25 (0.05)	**0.000**	0.17 (0.04)	0.16 (0.04)	**0.000**	0.18 (0.05)	0.16 (0.04)	**0.000**	−0.02 (0.06)/0.10 (0.09)/0.28 (0.07)/0.12 (0.08)/0.19 (0.09)	−0.02 (0.07)/0.10 (0.08)/0.27 (0.06)/0.11 (0.07)/0.19 (0.09)	0.735/0.228/**0.000**/0.117/**0.031**
Emotional support → Emotional support	0.26 (0.09)/0.35 (0.10)/0.01 (0.02)/0.01 (0.02)/0.01 (0.02)/	0.26 (0.08)/0.35 (0.09)/0.51 (0.08)/0.47 (0.08)/0.54 (0.07)	**0.002/0.000/0.000/0.000/0.000/**	0.39 (0.06)/0.39 (0.06)/0.39 (0.06)/0.39 (0.06)/0.39 (0.06)/	0.34 (0.05)/0.42 (0.07)/0.35(0.06)/0.43(0.09)/0.35(0.07)	**0.000/0.000/0.000/0.000/0.000**	0.33 (0.06)^a^	0.35 (0.06)	**0.000**	0.38 (0.04)^a^	0.39 (0.03)	**0.000**

Significant *p* values are represented in bold

*F* fathers, *M* mothers, *S* siblings, *FR* friends

^a^Parameters constrained to be equal over time. Stability paths that could not be constrained to be equal over time are separated by a slash; Wave 1 through Wave 6

The final multigroup models also had an acceptable fit with indices for RMSEA ranging between 0.01 and 0.04, for CFI between 0.89 and 0.99, and for TLI between 0.89 and 0.99. Stepwise releasing the stability paths across gender indicated that the stability paths of fathers’ emotional support (Δ*χ*^2^_SB_ (2) = 12.92, *p* = 0.002) and siblings’ prosocial behavior (Δ*χ*^2^_SB_ (1) = 15.47, *p* = < 0.001) differed for boys and girls. Furthermore, the correlation between the random intercepts of perceived autonomy support (Δ*χ*^2^_SB_ (1) = 46.79, *p* = < 0.001), and emotional support (Δ*χ*^2^_SB_ (1) = 18,59 *p* = < 0.001) from fathers and adolescents’ prosocial behavior differed between boys and girls. Moreover, the correlation between the random intercepts of perceived emotional support from siblings and adolescents’ prosocial behavior differed between boys and girls (Δ*χ*^2^_SB_ (1) = 4.00, *p* = 0.045). All other associations or lagged effects were equally strong for boys and girls.

### Between-Dyad Correlations

Between dyads, correlations showed that adolescents whose fathers, mothers and friends reported more prosocial behavior also reported more prosocial behaviors (see Table 3 for parents and Table 4 for friends). Associations between adolescents’ prosocial behavior and siblings’ prosocial behavior were nonsignificant (see Table 4).

**Table 3:** Associations of adolescents’ prosocial behavior with prosocial behavior, autonomy support and emotional support from fathers and mothers

	Father model			Mother model		
Parameters	*B* (SE)	*β* (SE)	*p*	*B* (SE)	*β* (SE)	*p*
Between-level correlations						
Prosocial behavior A with prosocial behavior F/M	0.04 (0.02)	0.19 (0.07)	**0.005**	0.04 (0.01)	0.17 (0.06)	**0.005**
Prosocial behavior A with autonomy support F/M	0.07 (0.01)/0.02 (0.01)	0.61 (0.08)/0.24 (0.13)	**0.000**/0.077	0.07 (0.01)	0.56 (0.08)	**0.000**
Prosocial behavior A with emotional support F/M	0.12 (0.02)/0.01 (0.02)	0.63 (0.08)/0.06 (0.09)	**0.000**/0.467	0.10 (0.02)	0.52 (0.08)	**0.000**
Within time associations T1						
Prosocial behavior A with prosocial behavior F/M	−0.00 (0.02)	−0.01 (0.07)	0.891	0.00 (0.02)	0.01 (0.05)	0.881
Prosocial behavior A with autonomy support F/M	0.07 (0.02)	0.24 (0.06)	**0.000**	0.06 (0.02)	0.25 (0.06)	**0.000**
Prosocial behavior A with emotional support F/M	0.09 (0.03)	0.23 (0.08)	**0.001**	0.09 (0.02)	0.26 (0.07)	**0.000**
Correlated change T2–T6						
Prosocial behavior A with prosocial behavior F/M	−0.00 (0.01)	−0.00 (0.01)	0.834	0.01 (0.01)	0.02 (0.02)	0.139
Prosocial behavior A with autonomy support F/M	0.03 (0.01)	0.14 (0.04)	**0.000**	0.04 (0.01)	0.17 (0.04)	**0.000**
Prosocial behavior A with emotional support F/M	0.03 (0.01)	0.08 (0.03)	**0.008**	0.04 (0.01)	0.26 (0.06)	**0.000**
Lagged-effects: adolescent effects^a^ T1–T6						
Prosocial behavior A → prosocial behavior F/M	0.01 (0.02)	0.01 (0.03)	0.681	0.02 (0.02)	0.04 (0.02)	0.104
Prosocial behavior A → autonomy support F/M	0.02 (0.02)	0.05 (0.03)	0.108	0.03 (0.01)	0.07 (0.03)	**0.027**
Prosocial behavior A → emotional support F/M	0.08 (0.05)	−0.03 (0.04)	0.440	0.03 (0.02)	0.05 (0.04)	0.567
Lagged-effects: parental effects^b^ T1–T6						
Prosocial behavior F/M → prosocial behavior A	0.07 (0.05)	0.03 (02)	0.124	0.05 (0.04)	0.03 (0.02)	0.246
Autonomy support F/M → prosocial behavior A	0.12 (0.07)	0.04 (0.03)	0.117	0.10 (0.06)	0.04 (0.02)	0.103
Emotional support F/M → prosocial behavior A	0.01 (0.05)	0.01 (0.03)	0.815	0.07 (0.07)	0.03 (0.03)	0.332

Separate models were conducted for autonomy support and emotional support, and for fathers, mothers, siblings and friends. T1 correlations show unconstrained within time fluctuations at T1. (un)Standardized within time associations and lagged effects were equal across time, hence represented by one value. Effects boys before the slash and effects girls after the slash. Significant *p* values are represented in bold

*A* adolescents, *F* fathers, *M* mothers

^a^Adolescents effects refer to lagged effects from adolescents’ prosocial behavior to autonomy or emotional support from fathers

^b^Parental effects refer to lagged effects from fathers’ or mothers’ prosocial behavior, autonomy/emotional support to adolescents’ prosocial behavior

**Table 4:** Associations of adolescents’ prosocial behavior with prosocial behavior, autonomy support and emotional support from siblings and friends

	Sibling model			Friend model		
Parameters	*B* (SE)	*β* (SE)	*p*	*B* (SE)	*β* (SE)	*p*
Between-level correlations						
Prosocial behavior A with prosocial behavior S/FR	0.03 (0.02)	0.13 (0.08)	0.120	0.06 (0.02)	0.33 (0.09)	**0.000**
Prosocial behavior A with autonomy support S/FR	0.04 (0.01)	0.32 (0.13)	**0.012**	0.06 (0.01)	0.58 (0.07)	**0.000**
Prosocial behavior A with emotional support S/FR	0.11 (0.02)/0.04(0.02)	0.42 (0.08)/0.20 (0.13)	**0.000**/0.116	0.11 (0.02)	0.56 (0.08)	**0.000**
Within time associations T1						
Prosocial behavior A with prosocial behavior S/FR	0.17 (0.04)	0.17 (0.06)	**0.004**	0.05 (0.04)	0.06 (0.05)	0.191
Prosocial behavior A with autonomy support S/FR	0.07 (0.02)	0.25 (0.07)	**0.000**	0.06 (0.02)	0.18 (0.06)	**0.000**
Prosocial behavior A with emotional support S/FR	0.11 (0.03)	0.28 (0.07)	**0.000**	0.09 (0.03)	0.18 (0.06)	**0.001**
Correlated change T2–T6						
Prosocial behavior A with prosocial behavior S/FR	0.02 (0.01)	0.04 (0.02)	0.053	0.01 (0.01)	0.02 (0.03)	0.450
Prosocial behavior A with autonomy support S/FR	0.03 (0.01)	0.10 (0.03)	**0.001**	0.04 (0.01)	0.13 (0.03)	**0.001**
Prosocial behavior A with emotional support S/FR	0.03 (0.01)	0.07 (0.03)	**0.006**	0.03 (0.01)	0.09 (0.03)	**0.005**
Lagged-effects: adolescent effects^a^ T1–T6						
Prosocial behavior A → prosocial behavior S/FR	0.03 (0.02)	0.03 (0.02)	0.166	0.04 (0.02)	0.04 (0.02)	0.091
Prosocial behavior A → autonomy support S/FR	0.04 (0.01)	0.07 (0.03)	**0.010**	0.02 (0.01)	0.05 (0.04)	0.192
Prosocial behavior A → emotional support S/FR	−0.03 (0.02)	0.08 (0.04)	0.071	−0.03 (0.02)	−0.04 (0.03)	0.249
Lagged- effects: S/FR effects^b^ T1–T6						
Prosocial behavior S/FR → prosocial behavior A	−0.02 (0.04)	−0.02 (0.04)	0.643	−0.00 (0.03)	−0.00 (0.02)	0.961
Autonomy support S/FR → prosocial behavior A	0.10 (0.07)	0.04 (0.03)	0.155	0.08 (0.06)	0.04 (0.03)	0.171
Emotional support S/FR → prosocial behavior A	0.03 (0.05)	0.02 (0.03)	0.574	0.03 (0.03)	0.02 (0.03)	0.418

Separate models were conducted for autonomy support and emotional support, and for fathers, mothers, siblings and friends. T1 correlations show unconstrained within time fluctuations at T1. (un)Standardized within time associations and lagged effects were equal across time, hence represented by one value. Significant *p* values are represented in bold

*A* adolescents, *S* siblings, *FR* friends

^a^Adolescents effects refer to lagged effects from adolescents’ prosocial behavior to autonomy or emotional support from friends

^b^S/FR effects refer to lagged effects from siblings’ or friends’ prosocial behavior, autonomy/emotional support to adolescents’ prosocial behavior

Adolescents who perceived more autonomy support from mothers, siblings, and friends also reported more prosocial behavior (see Table 3 for mothers and Table 4 for siblings and friends). Boys, but not girls, whose fathers were more supportive of their autonomy reported more prosocial behavior (see Table 3 for fathers). Moreover, the associations between adolescents’ prosocial behavior and emotional support from mothers and friends were significant (see Table 3 for mothers and Table 4 for friends). For fathers and siblings, this association varied across gender: Boys, but not girls, whose fathers and siblings were more emotionally supportive reported more prosocial behavior (see Table 3 for fathers and Table 4 for siblings).

### Concurrent Within-Dyad Associations

Concurrent associations between adolescents’ prosocial behavior and prosocial behavior of fathers, mothers, and friends were nonsignificant across all six measurements and invariant across gender (see Tables 3 and 4). At the first wave, siblings’ prosocial behavior was positively correlated with adolescents’ prosocial behavior. So, higher levels of siblings’ prosocial behavior during the first wave were related to higher levels of adolescents’ prosocial behavior during the first wave.

**Fig. 2:**
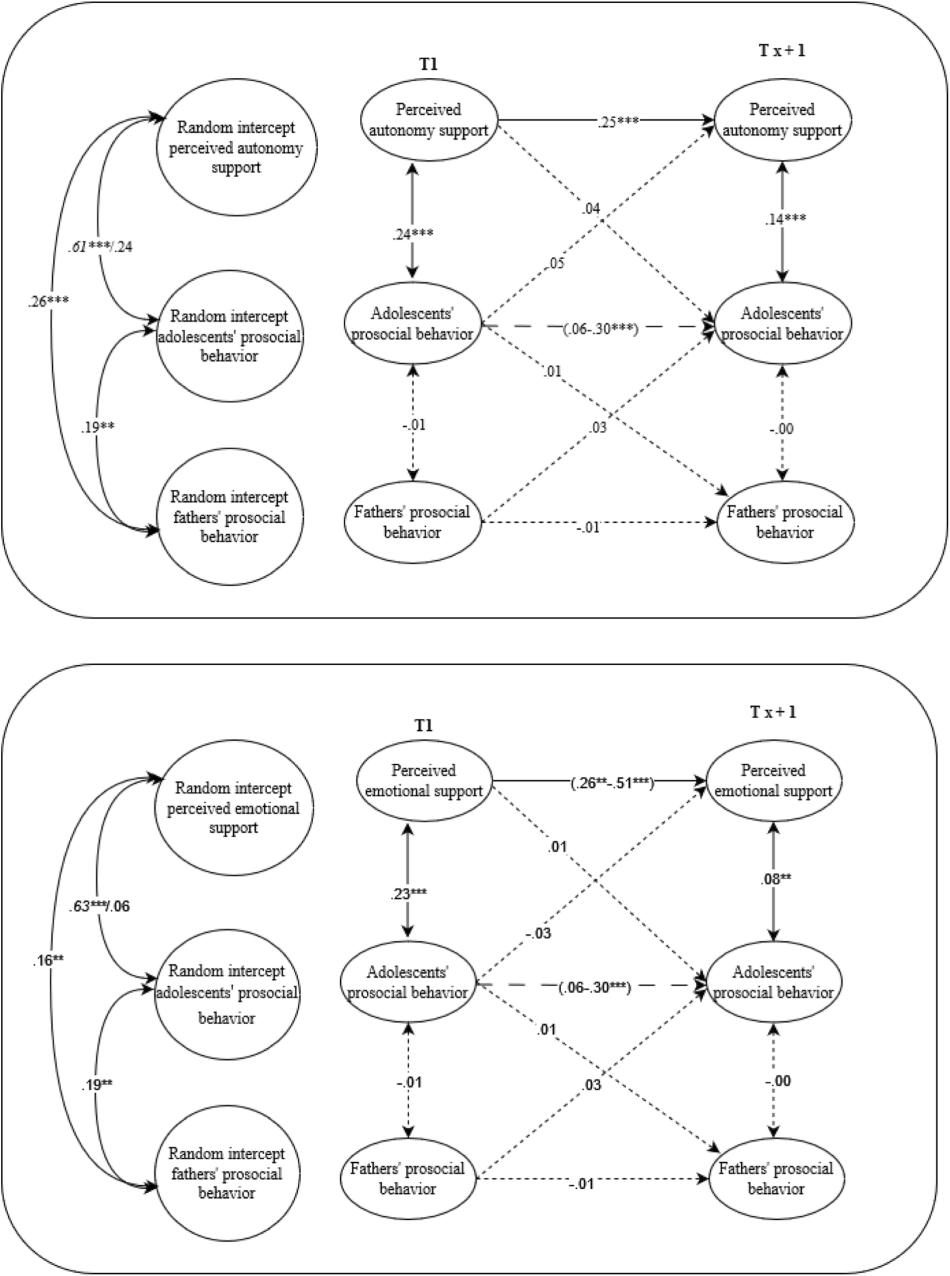
A visual representation of the final path models for adolescents and fathers. Random-intercept cross-lagged panel models on the associations between perceived autonomy support, perceived emotional support and prosocial behavior from fathers and adolescents’ prosocial behavior. Italic values before the slash represent boys and after the slash represents girls. Solid arrows represent relations that are significant, the dotted arrows represent relations that are not significant, the dashed arrows represent relations that could not be constrained over time and were at particular waves (non)significant. **p ≤ 0.01, ***p ≤ 0.001

**Fig. 3:**
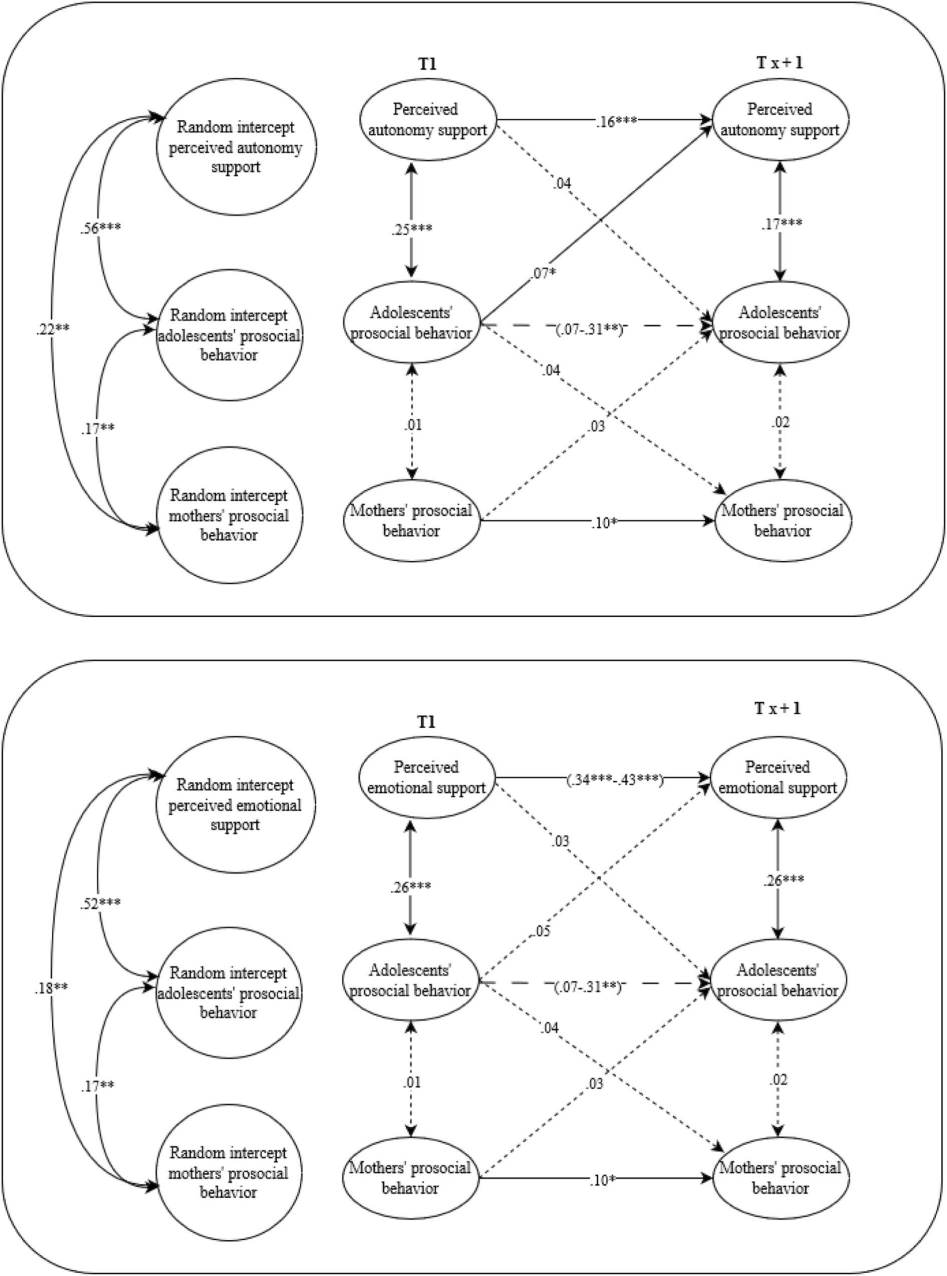
A visual representation of the final path models for adolescents and mothers. Random-intercept cross-lagged panel models on the associations between perceived autonomy support, perceived emotional support and prosocial behavior from mothers and adolescents’ prosocial behavior. Solid arrows represent relations that are significant, the dotted arrows represent relations that are not significant, the dashed arrows represent relations that could not be constrained over time and were at particular waves (non)significant. *p ≤ 0.05, **p ≤ 0.01, ***p ≤ 0.001

**Fig. 4:**
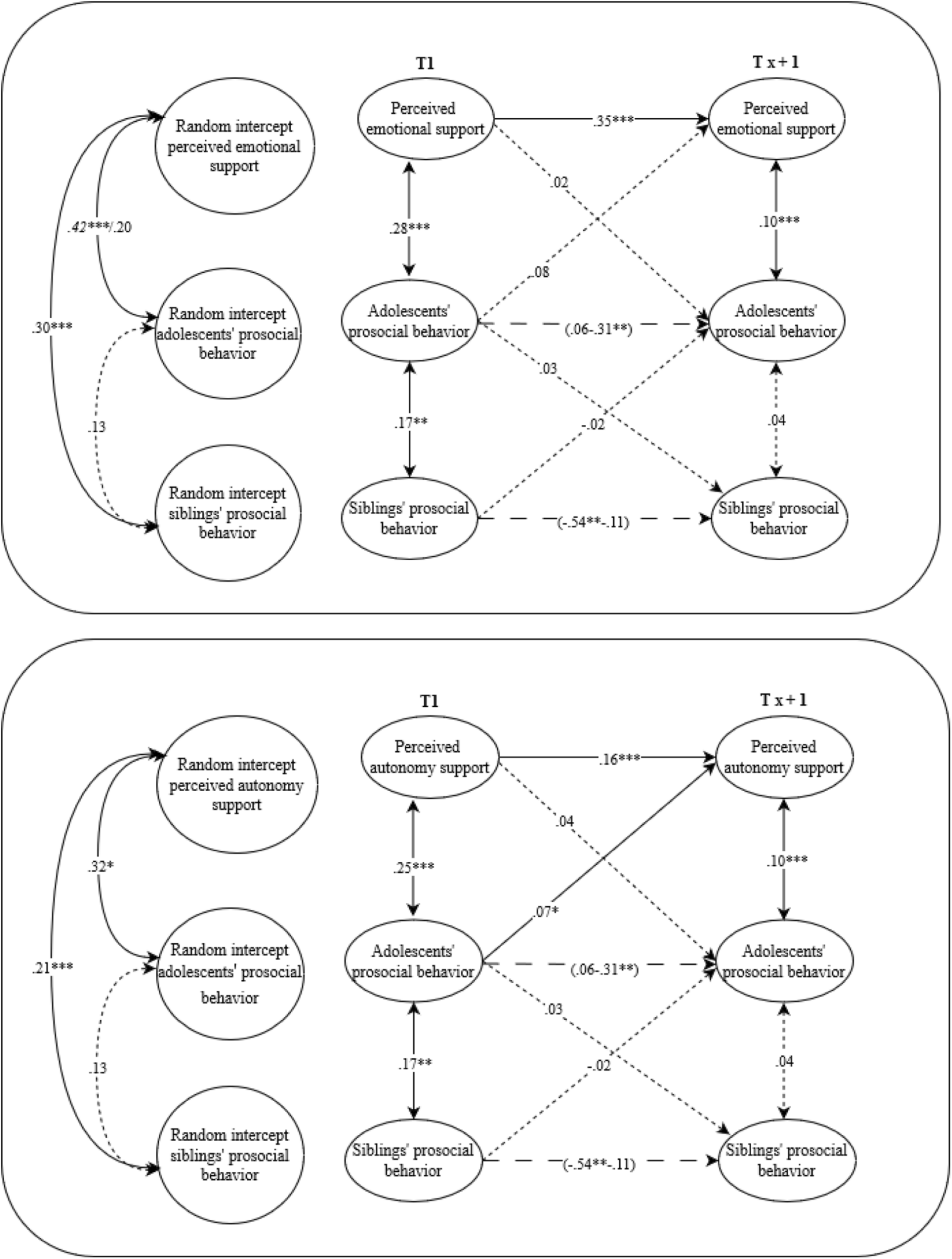
A visual representation of the final path models for adolescents and siblings. Random-intercept cross-lagged panel models on the associations between perceived autonomy support, perceived emotional support and prosocial behavior from siblings and adolescents’ prosocial behavior. Italic values before the slash represent boys and after the slash represents girls. Solid arrows represent relations that are significant, the dotted arrows represent relations that are not significant, the dashed arrows represent relations that could not be constrained over time and were at particular waves (non)significant. *p ≤ 0.05, **p ≤ 0.01, ***p ≤ 0.001

**Fig. 5:**
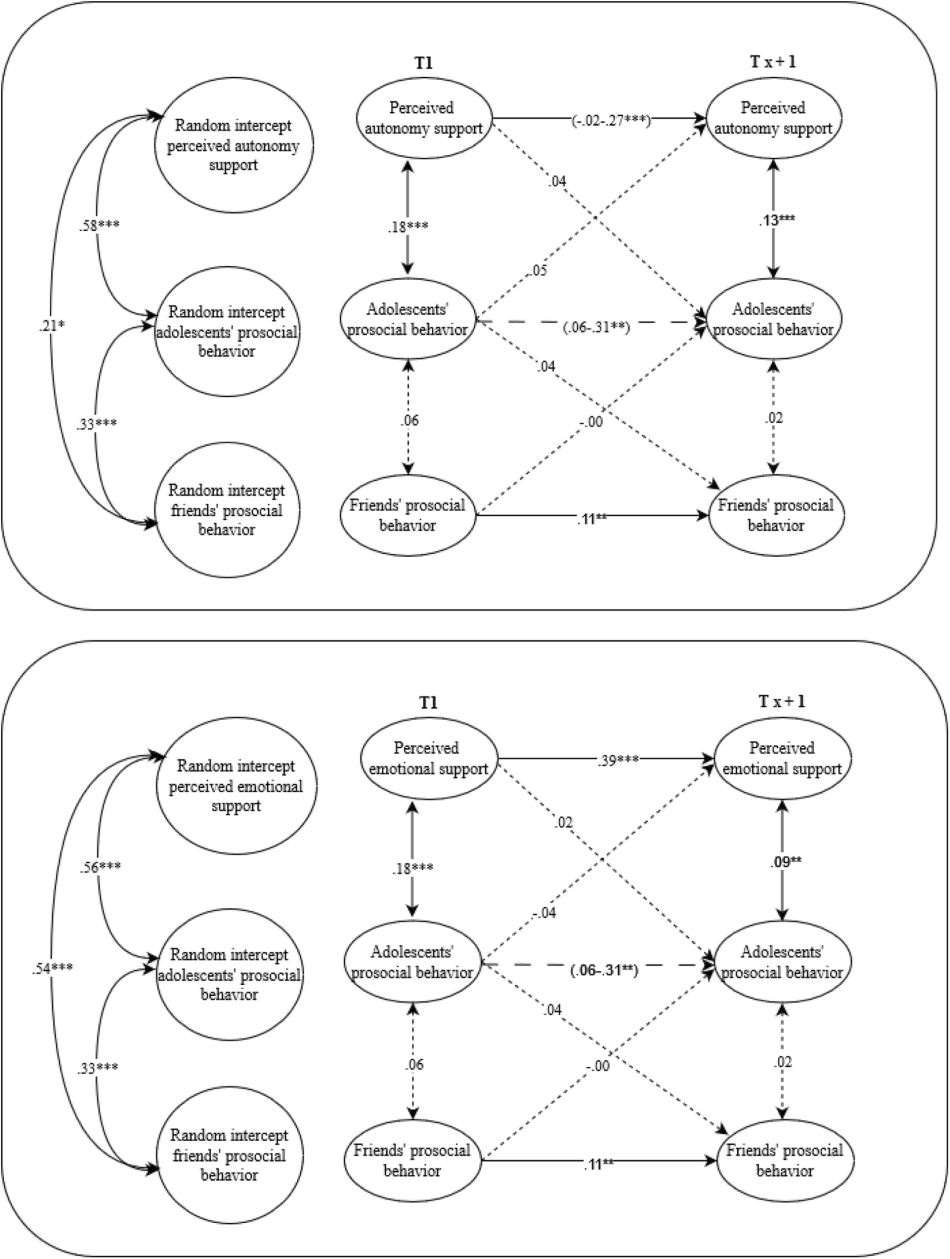
A visual representation of the final path models for adolescents and friends. Random-intercept cross-lagged panel models on the associations between perceived autonomy support, perceived emotional support and prosocial behavior from friends and adolescents’ prosocial behavior. Solid arrows represent relations that are significant, the dotted arrows represent relations that are not significant, the dashed arrows represent relations that could not be constrained over time and were at particular waves (non)significant. **p ≤ 0.01, ***p ≤ 0.001

The within-time associations of adolescents’ prosocial behavior with autonomy support and emotional support from fathers, mothers, siblings, and friends were consistently positively related, showing that when adolescents reported more prosocial behavior, they perceived more autonomy support within the same year (see Figs. 2–5).

### Longitudinal Within-Dyad Effects

For perceived autonomy support, we found that across all six waves, adolescents’ prosocial behavior predicted perceived autonomy support from mothers and siblings one year later (see Tables 3 and 4). Thus, when adolescents reported more prosocial behavior than usual, they perceived more autonomy support than usual from mothers and siblings in the subsequent year. Adolescents’ prosocial behavior was not significantly related to autonomy support from fathers and friends one year later (see Tables 3 and 4). Furthermore, perceived emotional support from fathers, mothers, siblings, and friends did not predict adolescents’ prosocial behavior. There were no significant effects between adolescents’ prosocial behavior and emotional support or prosocial behavior from fathers, mothers, siblings, and friends one year later.

### Sensitivity Analyses

Multigroup analyses were conducted to assess whether associations were different for younger versus older siblings. This was done by testing in a stepwise procedure whether model fit improved when parameters were allowed to vary between younger and older siblings. First, within-time correlations between perceived autonomy support or perceived emotional support from siblings and adolescents’ prosocial behavior and within-time correlations between siblings’ prosocial behavior and adolescents’ prosocial behavior were released. Second, the cross-lagged paths between perceived autonomy support or perceived emotional support, siblings’ prosocial behavior, and adolescents’ prosocial behavior were released. Third, between-dyad correlations between perceived autonomy or perceived emotional support, siblings’ prosocial behavior, and adolescents’ prosocial behavior were released. Each step was tested against the multigroup baseline model, in which all parameters were constrained to be equal for younger and older siblings.

Conducting the multigroup analyses revealed acceptable fit for all models (CFIs; 0.86–89; TLIs: 84–89; RMSEAs ≤0.080). Stepwise releasing the paths across younger and older siblings indicated that the cross lagged paths from adolescents’ prosocial behavior to perceived autonomy support from siblings (Δ*χ*^2^_SB_ (2) = 29.17, *p* < 0.001) differed between younger and older siblings. That is, within adolescent-sibling dyads adolescents’ prosocial behavior was consistently predictive of more perceived autonomy support from their younger siblings one year later (*b* = 0.08, *βs* = 0.12–0.20, *ps* ≤ 0.027), but not for their older siblings (*b* = 0.02, *βs* = 0.03–0.05, *ps* = 0.239–0.249). All other associations and lagged effects did not significantly differ between younger and older siblings.

## Discussion

Socialization influences from family and friends have consistent and important influences on adolescents’ prosocial behavior. Although theory suggests that it is essential to examine the combined effects of support and prosocial behavior in several interpersonal relationships, there is limited research testing these processes within dyads. To better understand the relations between support and prosocial behavior in adolescence, it’s important to conduct longitudinal studies that distinguish between within-dyad variance and between-dyad variance. The present six-year, multi-informant study investigated associations of adolescents’ prosocial behavior with prosocial behavior and perceived support from fathers, mothers, siblings, and friends both at the between-dyad level and within-dyad level. Almost all between-level associations were significant, indicating that adolescents with higher levels of prosocial behavior have family members and friends with higher levels of prosocial behavior and perceived more support from these family members and friends. Autonomy support and emotional support from family members and friends were consistently related to adolescents’ prosocial behavior at the same time point at the within-dyad level. Significant associations indicated that in periods when adolescents showed more prosocial behavior, they were surrounded by family and friends who were more supportive. The few significant longitudinal within-dyad effects indicated that adolescents’ prosocial behavior predicted perceived autonomy support from mothers and siblings one year later. The findings highlight the nuanced influences of fathers, mothers, siblings and friends in adolescents’ prosocial development.

### The Role of Prosocial Behavior From Family and Friends in Adolescents’ Prosocial Behavior

We expected that family and friends would serve as models, and therefore higher levels of their prosocial behavior would be associated with higher levels of adolescents’ prosocial behavior over time (e.g., Carlo et al., 2007). In line with the results of previous studies which used between-dyad designs (e.g., Carlo et al., 2007), we found that prosocial behavior of parents and friends was positively associated with adolescents’ prosocial behavior at the between-dyad level. However, results did not support that parents, siblings an friends serve as models for adolescents’ prosocial behavior over time. The absence of within-dyad associations and cross-lagged effects for parents, siblings and friends suggests that changes in parents’, siblings’ and friends’ prosocial behavior do not drive changes in adolescents’ prosocial behavior. An explanation for the lack of modeling effects could be that we used a global measure of prosocial behavior towards unspecified others. For example it is has been suggested that fathering and mothering are related to different targets and types of prosocial behavior (Padilla-Walker & Carlo, 2014). A study found that maternal warmth was positively related to prosocial behavior towards family members and mothers, whereas paternal warmth was positively associated with prosocial behavior towards fathers and friends (Padilla-Walker et al., 2016). Moreover, adolescents might be more inclined to show public prosocial behavior towards peers if they gain a more popular status in their peer group (Carlo & Padilla-Walker, 2020). It might be that taking a relational approach to the measurement of prosocial behavior, thus assessing prosocial behavior towards different targets, might yield distinct modeling effects in specific relationships.

The lack of lagged effects at the within-person level, suggests that the significant effects at the between-person level are explained by other factors than socialization. Genetic or epigenetic factors may explain some of the between person similarity. That is, genes from each person may contribute to the quality of interpersonal relationships (Kendler & Baker, 2007) as well as to the display of prosocial behaviors (e.g., Gregory et al., 2009), and environmental factors may also play a role in both prosocial behavior (Knafo Noam, et al., 2018) and support. Future studies should incorporate genetic and environmental factors as a third variable that can potentially explain why prosocial behavior and support are related at the between-person level.

### The Role of Interpersonal Support in Adolescents’ Prosocial Behavior

We expected that autonomy and emotional support from family members and friends would promote adolescents’ prosocial behavior (e.g., Hastings et al., 2007). Although we found positive associations on the between-dyad level and concurrent associations on the within-dyad level for fathers, mothers, siblings and friends, in contrast to our hypothesis, we did not find any lagged effects from autonomy support or emotional support to adolescents’ prosocial behavior. The absence of lagged within-dyad effects suggests that a change in support from parents or peers does not result in a significant change in adolescents’ prosocial behavior. This is in contrast to previous research that found longitudinal effects from fathers, mothers, siblings and friends on adolescents’ prosocial behavior (Harper et al., 2016; Wong et al., 2022), although prior research did not distinguish between-dyad variance from within-dyad variance. Hence, those findings may reflect stable between-person differences in support and adolescents’ prosocial behavior rather than longitudinal within-dyad processes.

Previous studies also found positive associations of autonomy support from parents (Gagné, 2003), friends (Ma et al., 2022) and from emotional support from parents (Wong et al., 2021), siblings (Harper et al., 2016) and from friends (e.g., Padilla-Walker et al., 2015b) with adolescents’ prosocial behavior. These positive associations might be explained by attachment theory (Mikulincer & Shaver, 2015), as adolescents who perceive their relationships with parents and peers as supportive likely have internalized prosocial standards and behaviors (Hastings et al., 2007). Perhaps positive internal working models are developed and become a context in which adolescents feel supported and accepted provides them with the resources to attend to others’ needs and display prosocial behaviors (Shaver et al., 2019).

Moreover, the concurrent associations within-dyads between perceived support and prosocial behavior suggest that these factors fluctuate together. This might indicate that adolescents are susceptible to short-term fluctuations in support from parents, siblings and friends but might also indicate that third factors affect both support perceptions and prosocial behavior. For example, economic strain or daily stressors might negatively affect prosocial behavior (Carlo et al., 2011) and might reduce support in the family (Conger et al., 1994). Further research might investigate whether within-dyads the link between parents’, siblings’ or friends’ prosocial behavior and adolescents is explained by third variables, such as daily stressors.

### The Role of Adolescents’ Prosocial Behavior in Interpersonal Relationships

We expected that adolescents’ prosocial behavior would promote autonomy support from parents, siblings and friends (e.g., Carlo et al., 2010; Meuwese et al., 2017), but results only showed some effects of adolescents’ prosocial behavior on perceived autonomy support. That is, increases in adolescents’ prosocial behavior promoted increases in perceived autonomy support in the relationships with their mothers and siblings in the subsequent year, but not in the relationships with fathers and friends. Adolescence is marked as a period in which children strive for more autonomy, which often goes together with increased conflicts, especially with mothers, as mothers and adolescents have different expectations about when they should be granted more autonomy (Burgoon, 2015). When adolescents show increased levels of prosocial behavior, this may reassure and convince mothers that their adolescents can handle increased independence and therefore mothers might be more supportive of adolescents’ autonomy. In contrast to our hypothesis, we did not find any effects on adolescents’ prosocial behavior to fathers’ autonomy support. Although there is a shift in caretaking responsibilities, the effects on maternal autonomy support might reflect the fact that mothers and adolescents are usually more often involved in daily hassles than fathers and adolescents.

Relationships with siblings provide a good context for practicing interaction behaviors due to their involuntary and horizontal nature as compared to friendships. The findings suggest that adolescents’ prosocial behavior might elicit more autonomy support from siblings than from friends. These findings could be due to the voluntary friendships which can dissolve at any time. That is, it might be that acting prosocial might be perceived as a more obligatory in sibling relationships but requires more intention and purpose to maintain relationships with friends. Thus, prosocial behaviors might have relatively more impact on autonomy support from siblings, rather than, friends.

In contrast to our hypothesis, adolescents’ prosocial behavior was not predictive of emotional support in any of the relational contexts. That we did find longitudinal within-dyad effects on autonomy support but not on emotional support might be because the affection that adolescents experience in their close relationships is not so much dependent on (changes in) adolescents’ behaviors and, in particular, their positive behaviors. Whereas increases or decreases in prosocial behavior may change adolescents’ perception of the autonomy support they receive, it likely takes more (for instance, severe or chronic problem behavior; Belsky, 1984) to elicit changes in family’s and friends’ love and affection for the adolescent.

### Gender Differences

We explored whether the links between adolescents’ prosocial behavior and prosocial behavior and support from parents and peers differed between boys and girls. Our results did not reveal much evidence for this notion. That is, at the within-dyad level all effects were invariant across gender. Only at the between-dyad level, associations between autonomy support and emotional support between fathers and adolescents’ prosocial behavior were significant for boys and not for girls, and the association between siblings’ autonomy support and adolescents’ prosocial behavior was significant only for boys, and not for girls. As the differences in effect sizes were not substantial, and as all other links were gender invariant, we conclude that there are no meaningful differences in parents’ and peers’ socialization of prosocial behavior of boys and girls in adolescence.

### Strengths and Limitations

This study comes with several limitations. First, the community sample consisted of adolescents from ethnic majority, intact families, and from families with a medium or high social economic status. Therefore, findings may not be generalizable to adolescents with different backgrounds. It may be that the role of family and friends in adolescents’ prosocial behavior is different for adolescents from families that have fewer resources. Future research should test these relations in a more diverse population. Secondly, we used annual measures, but since adolescence is a sensitive transitional period, it may be necessary to also include measures that are more closely spaced in time. Future research might consider micro or meso timescales to understand on what timescale adolescents’ prosocial behavior and autonomy support, emotional support, and prosocial behavior from fathers, mothers, siblings and friends interact. Autonomy support was defined as the promotion of volitional functioning, and is a multidimensional construct consisting of acknowledging and respecting adolescents’ feelings, giving a rationale for rules and demands, and providing choices and opportunities for initiative taking (Joussemet et al., 2008). The scale used in the current study mainly measures the extent to which parents, siblings and friends provide opportunities for initiative taking and acknowledge and respect adolescents’ decisions and feelings (e.g., McCurdy et al., 2020). However, the used scale did not measure providing rationale and explanation for rules, limits and demands. This domain might also be important predictors of adolescents’ prosocial behavior. Further research is encouraged to include questionnaires that assess all different autonomy supportive behaviors, and to discern which types of autonomy support are associated with different types of prosocial behaviors.

Notwithstanding the limitations, this study has multiple strengths. First, the study covered six years, which enabled us to identify (the direction of) links across adolescence. Second, we included reports from adolescents, parents, siblings, and friends on their own prosocial behavior and adolescents’ perception of support from different fathers, mothers, siblings and friends. However, adolescents’ prosocial behavior and all autonomy and emotional support variables were based on adolescent reports, therefore some associations among these constructs might be inflated due to shared method variance. Third, the study was pre-registered, enabling transparency in how the study was conducted and in testing our hypotheses instead of exploring datasets. Fourth, we separated between-person variance from the within-person variance. In doing so, we showed that the positive links between adolescents’ prosocial behavior, and support and prosocial behavior from fathers, mothers, siblings and friends that were found in previous research are probably based on differences between adolescents and families and friends rather than processes within-dyads where adolescents, families and friends affect each other.

## Conclusion

To better understand the relation between support and prosocial behavior in adolescence, it is important to conduct longitudinal studies that distinguish between within-dyad variance and between-dyad variance, and to study this across different relationship types. The results showed that almost all associations of support and prosocial behavior of family and friends with adolescents’ prosocial behavior were significant at the between-dyad level. As previous research did not investigate the separate roles of fathers, mothers, siblings and friends systematically across adolescence, the current study contributed to the literature by showing that mothers’, fathers’, siblings’ and friends’ prosocial behavior and support is indeed positively associated to adolescents’ prosocial behavior. In sum, this study identified associations between within-dyad changes in prosocial behavior and support. However, there was no support for lagged effects from prosocial behavior from fathers, mothers, siblings and friend to adolescents’ prosocial behavior. The current study used annual reports, it might be that examining the direction of effects using a different time scale might yield different effects. Since adolescence is an dynamic period marked by a lot of change it might be that changes in support and prosocial behavior occur on a different timescale. Future research might use different timescales in longitudinal designs to understand on what timescale adolescents’ prosocial behavior and autonomy support, emotional support, and prosocial behavior from fathers, mothers, siblings and friends are interconnected. The study highlights the importance of using RI-CLPM models to investigate the role of parents and peers in the socialization of adolescents’ prosocial behavior. Such research can provide more accurate assessments of the links between prosocial behavior and support from fathers, mothers, siblings and friends and adolescents’ prosocial behavior.

### Supplementary information

Supplementary Information
