# Supplementary material for: Longitudinal Associations Between Support and Prosocial Behavior Across Adolescence: The Roles of Fathers, Mothers, Siblings, and Friends
Source: J Youth Adolesc. 2024 Jan 20;53(5):1134–54. doi: 10.1007/s10964-023-01885-5 (PMC10980607; doi:10.1007/s10964-023-01885-5)
Supplement: Supplementary file 1 — Supplementary Information [file 10964_2023_1885_MOESM1_ESM.docx]

# Supplementary Information

**Appendix A**

| Table S1  *Associations of Fathers’, Mothers’, Siblings and Friends’ Prosocial Behavior with Their Autonomy Support and Emotional Support* | | | | | | | | | | | | | | | |
| --- | --- | --- | --- | --- | --- | --- | --- | --- | --- | --- | --- | --- | --- | --- | --- |
| Model | Father model |  |  | Mother Model |  |  | Sibling Model |  |  | Friend Model |  | |  | |  |
|  | *B (SE)* | β *(SE)* | *p* | *B (SE)* | β *(SE)* | *p* | *B (SE)* | β *(SE)* | *p* | *B (SE)* | β *(SE)* | | *p* | |  |
|  |  |  |  |  |  |  |  |  |  |  |  | |  | |  |
| **Between-level correlations** |  |  |  |  |  |  |  |  |  |  |  | |  | |  |
| Prosocial behavior F/M/S/FR with autonomy support F/M/S/FR | .03 (.01) | .26 (.06) | **.000** | .03 (.01) | .22 (.06) | **.005** | .04 (.01) | .21 (.06) | **.001** | .02 (.01) | .21 (.09) | | **.027** | |  |
| Prosocial behavior F/M/S/FR with emotional support F/M/S/FR | .01 (.01) | .16 (.06) | **.008** | .03(.01) | .18 (.07) | **.009** | .10 (.02) | .30 (.07) | **.000** | .08 (.02) | .54 (.10) | | **.000** | |  |
| **Within time correlations T1** |  |  |  |  |  |  |  |  |  |  |  | |  | |  |
| Prosocial behavior F/M/S/FR with autonomy support F/M/S/FR | .00 (.01) | .07 (.06) | .304 | -.02 (.01) | -.14 (.06) | **.018** | .01 (.02) | .02 (.06) | .701 | .03 (.02) | .08 (.05) | | .135 | |  |
| Prosocial behavior F/M/S/FR with emotional support F/M/S/FR | .02 (.01) | .01 (.07) | .917 | -.01 (.01) | -.06 (.06) | .320 | .01 (.02) | .02 (.05) | .623 | .01 (.03) | .02 (.05) | | .770 | |  |
| **Correlated change T2-T6** |  |  |  |  |  |  |  |  |  |  |  | |  | |  |
| Prosocial behavior F/M/S/FR with autonomy support F/M/S/FR | -.01 ^a^ (.00) | -.03 (.03) | .287 | .01 (.01) ^a^ | .02(.02) | .444 | .00 (.00) ^a^ | .01 (.02) | .494 | .01 (.01) ^a^ | .05 (.03) | | .085 | |  |
| Prosocial behavior F/M/S/FR with emotional support F/M/S/FR | .02(.01) | .10 (.08) | .206 | .01 (.01) | .03 (.02) | .066 | -.01 (.01) | -.04 (.02) | .127 | .02 (.01) | .04 (.03) | | .180 | |  |
| **Lagged effects T1-T6** |  |  |  |  |  |  |  |  |  |  |  | |  | |  |
| Prosocial behavior F/M/S/FR 🡪 emotional support F/M/S/FR | -.04 (.04) ^a^ | -.03 (.03) | .394 | .02 (.03) ^a^ | .02 (.03) ^a^ | .567 | -.02 (.02) ^a^ | -.04 (.03) | .250 | .02 (.02) ^a^ | .02 (.03) | | .411 | |  |
| Autonomy support F/M/S/FR 🡪 prosocial behavior F/M/S/FR | -.06 (.04) ^a^ | -.05 (.03) | .114 | -.05 (.09)/  -.08 (.08)/  .04 (.06)/  .18 (.06)/  .03 (.06) | -.03 (.06)/  -.06 (.06)/  .04 (.06)/  .17 (.05)/  .03 (.06) | .594/  .262/  .505/  .001/  .994 | -.01 (.05) ^a^ | -.01 (.05) | .888 | .01 (.05) ^a^ | .01 (.03) | | .783 | |  |
| Emotional support F/M/S/FR 🡪 prosocial behavior F/M/S/FR | -.01 (.03) ^a^ | - .01 (.04) | .806 | .06 (.04) ^a^ | .05 (.03) ^a^ | .074 | -.05 (.04) ^a^ | -.03 (.03) | .250 | .01 (.04) ^a^ | .01 (.03) | | .829 | |  |
| *Note.*  F = Fathers; M=Mothers; S= Siblings; F= Friends. ^a^ = parameters constrained to be equal over time. Cross-lagged effects and associations that could not be constrained to be equal over time are separated by a slash; Wave 1 through Wave 6. Significant p-values are represented in bold. | | | | | | | | | | | |  | |  | |

Supplementary information

“Few studies using the same data as the current study have examined the associations of autonomy support with educational identity formation (Doeselaar et al., 2015), problem behavior (Crocetti et al., 2016a; van der Giessen et al., 2014; Vrolijk et al., 2020), emotion regulation difficulties (Keskin & Branje, 2022), and emotional variability in in parent-adolescent interaction (van der Giessen et al., 2015). In addition, emotional support has been found to be related to depressive symptoms (Hale et al., 2019; Buist et al., 2019), and externalizing problems (Crocetti et al., 2016a). These studies showed small to moderate effect sizes. Regarding prosocial behavior, previous studies using the same data have examined the mean level development of prosocial behavior and the associations with empathy in adolescence (Van der Graaff et al., 2018). Furthermore, prosocial behavior has been examined in relation to self–concept clarity longitudinally (Crocetti et al., 2016b). Moreover, most studies in the broader literature (utilized other data sets) showed that negative behaviors, such as adolescents’ adjustment problems are negatively related to adolescents’ prosocial behavior with small effect sizes (Padilla-Walker et al., 2018).”
